# Gall-Colonizing Ants and Their Role as Plant Defenders: From ’Bad Job’ to ’Useful Service’

**DOI:** 10.3390/insects10110392

**Published:** 2019-11-06

**Authors:** Daniele Giannetti, Cristina Castracani, Fiorenza A. Spotti, Alessandra Mori, Donato A. Grasso

**Affiliations:** Department of Chemistry, Life Sciences & Environmental Sustainability, University of Parma, 43124 Parma, Italyfiorenzaaugusta.spotti@unipr.it (F.A.S.); alessandra.mori@unipr.it (A.M.); donato.grasso@unipr.it (D.A.G.)

**Keywords:** ant–plant interactions, mutualism, oak-gall secondary fauna, nest architecture, indirect plant defence

## Abstract

Galls are neoformed structures on host plant tissues caused by the attack of insects or other organisms. They support different communities of specialized parasitic insects (the gall inducers), and can also provide refuge to other insects, such as moths, beetles and ants, referred to as secondary occupants. This study focuses on galls induced by the oak gall wasp *Andricus quercustozae* and secondarily colonized by ants in a mixed oak forest. A field survey and two experiments were carried out to a) study ant (species-specific) preferences for different features of the galls, b) describe differences in gall architecture due to ant activity, c) analyse the effects of the presence of gall-dwelling ants on plant health. The results show that there are differences between ant species in gall colonization and in the alteration of gall opening and inner structure. We verified that gall-dwelling ants protect their host plants efficiently, offering them an indirect defence mechanism against enemies (predators and pathogens). The data suggest a new paradigm in ant–plant relationships mediated by the presence of galls on the plants whose ecological and evolutionary implications are discussed.

## 1. Introduction

Galls are neoformed structures on host plant tissues caused by the parasitic action of insects or other organisms [1,2,3,4,5]. In the 19th century, many biologists assumed that plants controlled the formation of galls [6]. It is now commonly believed that galls are induced by parasites, although in many cases, the mechanism of formation is not known [6,7].

The global richness of gall-inducer insects is estimated at about 133,000 species [4,8,9]. In particular, gall wasps (Hymenoptera: Cynipidae) constitute a relevant group of gall-inducing organisms, with roughly 1400 described species [6]. They mainly prefer a range of oak species as hosts, but the use of other plants is also described (e.g., trees of the *Fagaceae* family and herbs of the *Asteraceae, Lamiaceae, Rosaceae* and *Papaveraceae* families) [6,10]. Gall wasps are mainly distributed in the temperate zone of the Northern Hemisphere, with around 1000 species falling into 25 genera. In particular, the highest species diversity is found in the Nearctic region, with an estimated 700 species [7,11].

Galls can also be considered as the product of ecosystem engineering by several types of organisms [12,13,14,15]. Apart from gall inducers, abandoned (or senescent) galls can provide refuge for other arthropods, such as spiders, insects and myriapods. Among insects, many orders are described, with Coleoptera and Lepidoptera being the most represented [16,17,18]. Tόrossian [16] refers to these inhabitants as “secondary fauna” divided into arboreal or terrestrial according to the position of the gall. Abandoned galls usually represent a temporary or permanent shelter for larvae and/or adults, thereby modifying the local community structure and playing an important role in facilitative interactions [19].

The gall secondary fauna also includes a number of ant species belonging to different subfamilies and widespread in several habitats [18,20,21,22,23]. Tόrossian, [20,21] found that ant species in temperate environments differed in their choice of galls to colonize according to the position of galls on plants, whereas in tropical areas, gall size seemed to play a major role [23]. In both cases, the results showed that galls could host colonies at different life stages or with different compositions (e.g., only queen, only adults, adults and brood, or adults, brood and queen).

The role of galls in facilitating ant colonization of plants can has interesting positive effects on plant defence and consequently, plant health. In fact, the role of ants as plant defenders has been well established in several contexts [24,25,26,27,28,29,30]. As shown by Rosumek et al. [25], the defensive effects of ants can be the reduction in herbivore abundance, and species diversity and herbivory rates [31,32,33]. For example, ants can reduce the number of herbivores, such as termites [34] and lepidopteran larvae [35]. As a consequence, ants can promote the increase in plant biomass and leaf-flower-fruit-seed production, ultimately affecting plant fitness [36,37]. Moreover, in their meta-analysis, Chamberlain and Holland [38] showed that ant effects on plants are not generally context-dependent, but are instead routinely positive, resulting in a reduction in herbivory and an increase in plant performance. In Italy, research on galls dates back to the 19th century, mainly concerning systematics and distribution of gall inducers and plant hosts in Sicily [39,40]. More recently, interest in this topic has grown among Italian researchers, focusing on gall inducers, their distribution and classification, and on their control when considered as agricultural pests [41,42,43]. However, records about secondary fauna, and in particular, detailed analyses on gall colonization by ants and its consequences on the host plant are still lacking.

The aim of this study was to investigate features and implications of gall colonization by ants using *Andricus quercustozae* oak galls as a model system in the study of ant–plant relationships. In this context, the goals of this study were *i.* the checklist of ant species and the evaluation of species-specific differences in gall colonization, *ii.* the analysis of gall secondary architecture due to ant excavation and building, *iii.* the analysis of the defensive effects on the host plant due to ant presence. With regard to these aims, we expected i. to compile the first Italian checklist of gall-colonizing ants and to highlight gall preference by the different ant species, ii. to point out and describe for the first time species-specific patterns of gall modification by ants, iii to demonstrate that ants can have positive effects on their host plant health.

## 2. Materials and Methods

### 2.1. Study Area and Gall Selection

The field survey and the experiments were conducted in the Lunigiana area located in Northern Tuscany (Italy) near the village of Fornoli-MS in a mixed oak forest. Galls were located on *Quercus* spp. trees and induced by *Andricus quercustozae* (Hymenoptera: Cynipidae). This species has a cycle that involves host alternation. The sexual generation develops in galls induced in plants of *Cerris* section, while parthenogenetic generation is found only in galls of *Quercus sensu stricto* [7,44,45]. In this study area, oaks had a variable number of galls from 2 to 23.

### 2.2. Gall Colonisation

#### 2.2.1. Field Survey

**Figure 1:**
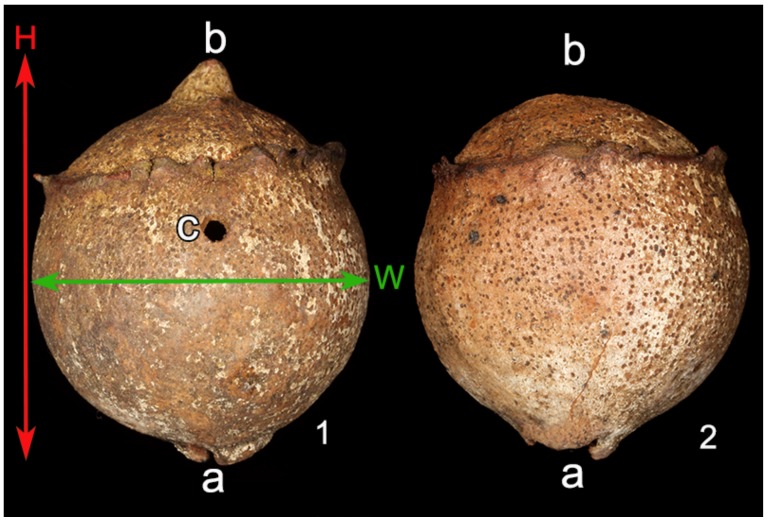
Linear dimensions measured on the gall and cutting procedure. The vertical line (H) shows Height measured as the distance between the insertion point of the gall on the tree branch (a) and the opposite peak (b). The horizontal line (W) shows the width, measured as the longest perpendicular line to the height. Each gall was divided into two halves (1,2) according to the height line (H), keeping the galler’s hole (c) in the middle of one of the halves.

A field survey was conducted in October and November 2016. The gall samples were collected in a 300 m^2^ area; they bore the typical oak gall wasp’s hole, showing that they had already been abandoned by the gall-inducer. Before gall collection, its height from the ground (named below as “position on the plant”) was measured. In the laboratory, all the galls were checked and excluded from further analyses whenever they had damages preventing further measurements or they hosted arthropods other than ants (e.g., spiders or beetles). The remaining galls were measured according to two linear dimensions, i.e., height, defined as the distance between the insertion point of the gall on the tree branch and the opposite peak, and width, measured as the longest perpendicular line to height (Figure 1). Each gall was then cut into two halves according to the height line keeping the galler’s hole approximately in the middle of one of the halves (Figure 1). The presence of ants was recorded and individuals were classified as queens, workers and brood. Ants were sorted and identified to species level. Identification was achieved in collaboration with Dr. Enrico Schifani (Myrmecology Lab, University of Parma) and Dr. Fabrizio Rigato (ant taxonomist at the Natural History Museum of Milan, Italy).

In order to investigate differences between ant species, One-way ANOVA tests were run on the three variables measured: 1. position on the plant; 2. gall height; 3. gall width. Species were set as factor, and Tukey Post Hoc tests were performed when necessary.

For *Crematogaster scutellaris,* the most abundant species (see Results section), gall categories were created according to ant colony composition: 1. galls with queens only (Cs-Q), 2. galls with queens, workers and brood (Cs-qwb), 3. galls with workers and brood (Cs-wb), 4. galls with workers only (Cs-w). These categories were used as factor in One-way ANOVA tests in order to find differences in gall position on the plant and size (height and width).

#### 2.2.2. Experiment 1

A long-term field experiment had been conducted starting in October and November 2015, when a set of 187 galls was selected in the same area as the field survey. The galls did not have the typical oak gall wasp’s hole, meaning that the cynipid was still inside. These galls were isolated by means of a safety net preventing ant or other arthropod colonization once the wasp emerged. In October and November 2016, the galls were scanned and 76 were excluded because they presented at least one of the following conditions: i) no oak gall wasp’s hole was detected, suggesting that the gall-insect development was aborted and the gall was no longer colonisable; ii) evidence of net damages and ant or other arthropod colonization; iii) clearly damaged galls. The remaining 111 galls were used for the experiment and the net was removed. One year later (October and November 2017), the galls were collected so that all had been exposed to possible ant colonization for the same length of time. During collection, the height from the ground (named below as “position on the plant”) was measured. None of the experimental galls appeared to be damaged, so they were all used to measure their size, using the same methods as described above (see Figure 1). Each gall was then divided into two halves along to the height line keeping the galler’s hole approximately in the middle of one of the halves (Figure 1). The content was subsequently evaluated by recording the ant species possibly present and the colony composition (queens, workers and brood). In this case, the ants were also sorted and identified at species level. To investigate differences between ant species, statistical analyses were performed as described above (see 2.2.1).

For *Cr. scutellaris*, the most abundant species (see Results section), gall categories were created according to colony composition and statistical analyses were run as described above (see 2.2.1).

### 2.3. Nest Architecture

We analysed the inner architecture of the galls due to ant excavation. As a first step, the two halves of the galls were qualitatively described according to the shape and position of the hole of excavation, the proportion of removed material and the presence of peculiar structures. Galls were classified according to ant species colonization (*Temnothorax* spp., *Colobopsis truncata, Cr. scutellaris* and empty). Galls colonized by *Cr. scutellaris* were further divided into categories according to ant colony composition (Cs-q; Cs-qwb; Cs-wb; Cs-w). The galls used for the analysis were a subset of those sampled during Experiment 1. A total of 56 galls were used: 8 galls for each of the 7 previous categories (species colonization and colony composition).

A second step of the analyses was performed on the same galls to evaluate the proportion of removed material. A 2D excavation area image was obtained for each half of each gall by using the stereomicroscope Zeiss Stemi 508, the Axiocam Erc 5s and a focus stacking technique. The area of excavation was measured using the Zeiss Zen core Software. The effect of gall categories on the excavation area was tested using One-way ANOVA and Tukey post hoc tests.

Thirdly, the excavation volume of empty galls and of those colonized by *Cr. scutellaris* was assessed using internal moulds. The galls used for this analysis were the ones collected during Field Experiment 1. A mixture of alginate and water was poured inside each half of the gall to make a mould. After 24 h, the gall residue was removed. Each gall mould was assembled and stuck together with glue. Finally, the excavation volume was determined by immersing the mould in a tube (Ø= 40 mm) filled with a known volume of water (V = 15 ml) and calculating the increase in the water volume. Each gall was classified as either empty or according to the 4 categories of ant colony composition; their effect on excavation volume was tested with a One-way ANOVA test and Tukey post hoc tests.

### 2.4. Defensive Effect of Ants on Plants: Experiment 2

In May 2017, 48 young plants (*Quercus* spp.) were selected in an area (100 m^2^) close to the collection field station (see above). These plants were at least one year old, with 4–7 leaves each and with a relative distance of at least 1 m. On each of the 24 plants, we added 1 gall carrying *Cr. scutellaris* ants. The galls were collected from older oak trees in the same area and manipulated in order to induce the ants to exit in order to be able to count them. Only galls with at least 80 ants were used for the experiments. The other 24 plants carried 1 empty gall each and were isolated with adhesive strips at the base of the trunk to prevent ant colonization. From May to August 2017, 24 scan samplings were used to record the presence of possible phytophagous insects counting the number of Lepidoptera (caterpillars and adults) and Coleoptera (adults) and recording the presence of damage typically produced by leaf-miners (Diptera or Lepidoptera). Moreover, at the end of the experiment (August 2017), the number of leaves damaged by chewing insects and the number of leaves attacked by fungi were counted. The experiment was repeated on another set of 48 plants in May–August 2018.

A total of 76 plants was included in the analysis as 11 plants from 2017 and 9 plants from 2018 were excluded since they were either dead or ant-free by the end of the experiment. We used Two-way ANOVA tests to verify the effects of year (2017 vs. 2018) and the presence of ants (with ants vs. without ants) on the following variables:

1. PI: Number of phytophagous insects counted in all the 24 samplings (only caterpillars of Lepidoptera and adult of Coleoptera were present)
2. CI: the proportion of leaves attacked by chewing insects on the total leaves in each plant at the end of the experiment.
3. FU: the proportion of leaves attacked by fungi on the total leaves in each plant at the end of the experiment.
4. LM: the proportion of leaves attacked by leaf miners on the total leaves in each plant at the end of the experiment.

For CI, FU and LM, the data were transformed according to the following formula: X = arcsin (sqr x), where x is the calculated variable.

In 2.2, 2.3 and 2.4, all the statistical analyses were performed using IBM statistical software SPSS 20.0 for Windows package.

## 3. Results

### 3.1. Gall Colonisation: Field Survey and Experiment 1

After the preliminary scan, 100 galls from the field survey were selected and used to evaluate their size and content. The results of the content analysis show that 74 galls were colonized by ants (see Table 1), 21 were empty, four mouldy and one colonized by a beetle (Coleoptera). *Cr. scutellaris* and *Co. truncata* showed more than one type of colony composition, while a complete colony with queen and larvae was recorded for all the other species (except *Dolichoderus quadripunctatus*) (see Table 1).

We excluded the categories with less than five samples from the analysis (mouldy galls and galls with beetle, with *Camponotus* spp., *D. quadripunctatus*, *Cr. scutellaris nigra*) in order to investigate differences between ant species concerning gall position on plant and gall size. In addition, we merged data from the species of the genus *Temnothorax* into a single group (*Temnothorax* spp.).

**Figure 2:**
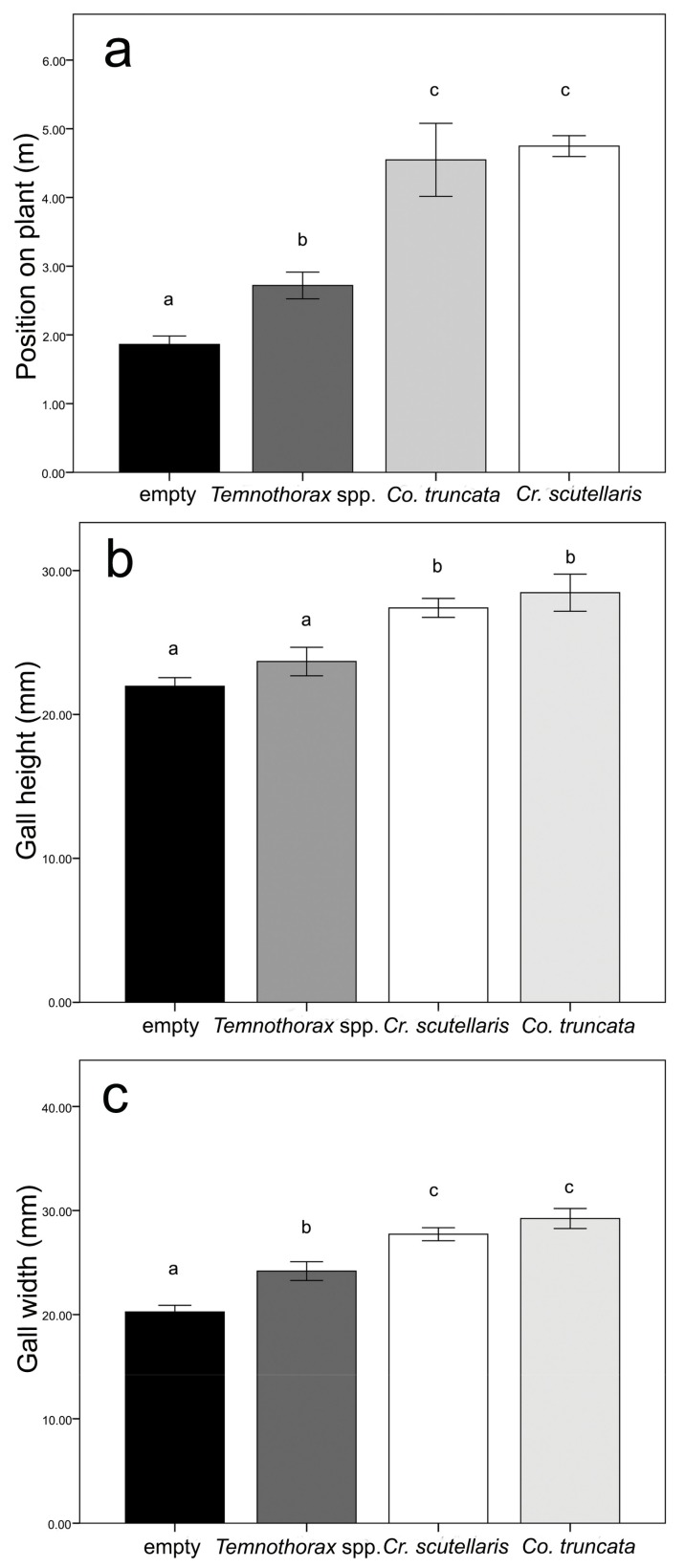
Graphs showing gall preferences of the different ant species according to the data collected in the field survey. The gall preferences are measured as gall position on plant (**a**), gall height (**b**), and gall width (**c**). The SE interval is shown for each bar. The bars with the same letter are not statistically different (One-way ANOVA, see text for further details).

The statistical analyses showed significant differences in gall size and position on the plant according to ant species (n = 88; Position: F_3,84_ = 52.26 *P* < 0.001; Height: F_3,84_ = 12.26 *P* < 0.001; Width: F_3,84_ = 23.76 *P* < 0.001). With regard to the position on the plant, the Tukey tests (Table S1) showed three different groups with the highest values for *Cr. scutellaris* and *Co. truncata*, the lowest values for empty galls, and values for *Temnothorax* spp. galls in between (Figure 2a). As for gall size parameters, Tukey test comparisons of gall height (Table S1) showed two groups, the biggest galls being colonized by *Cr. scutellaris* and *Co. truncata*, and the smallest by *Temnothorax* spp. or empty (Figure 2b). With regards to gall width, post-hoc tests (Table S1) revealed three groups with the highest values for *Cr. scutellaris* and *Co. truncata* galls, the lowest values for empty galls, and values for *Temnothorax* spp. galls in between (Figure 2c).

The analysis of colony composition in the galls colonized by *Cr. scutellaris* (n = 39) showed that three galls hosted single queens only, 14 galls a queen, workers and brood (No. of workers: Mean ± SE 101.9 ± 7.9), 19 galls workers and brood (No. of workers: 185.2 ± 34.9), and three galls workers only (No. of workers: 493.0 ± 81.67). In order to perform the statistical analysis, the galls were divided into 2 groups: galls with the queen and galls without the queen. The one-way ANOVA tests showed that there are differences in gall size according to the presence of the queen (Height: F_1,37_ = 17.49, *P* < 0.001; Width: F_1,37_ = 19.41, P < 0.001). The galls with the queen were smaller than the ones without (Mean ± SE—Height: with queen 24.9 ± 0.9 mm, without queen 29.6 ± 0.7 mm; Width: with queen 25.2 ± 0.6 mm, without queen 30.0 ± 0.8 mm). No statistical differences were found for the position on the plant (F_1,37_ = 1.96, P = 0.169; Mean ± SE: with queen 4.6 ± 0.2 m, without queen 5.0 ± 0.2 m).

As for data from Experiment 1, the results of the content analysis show that 94 experimental galls were colonized by ants (see Table 1), 16 were empty and one mouldy. *Cr. scutellaris* and *Co. truncata* showed more than one type of colony composition, while only one type was recorded for all the other species (see Table 1).

**Figure 3:**
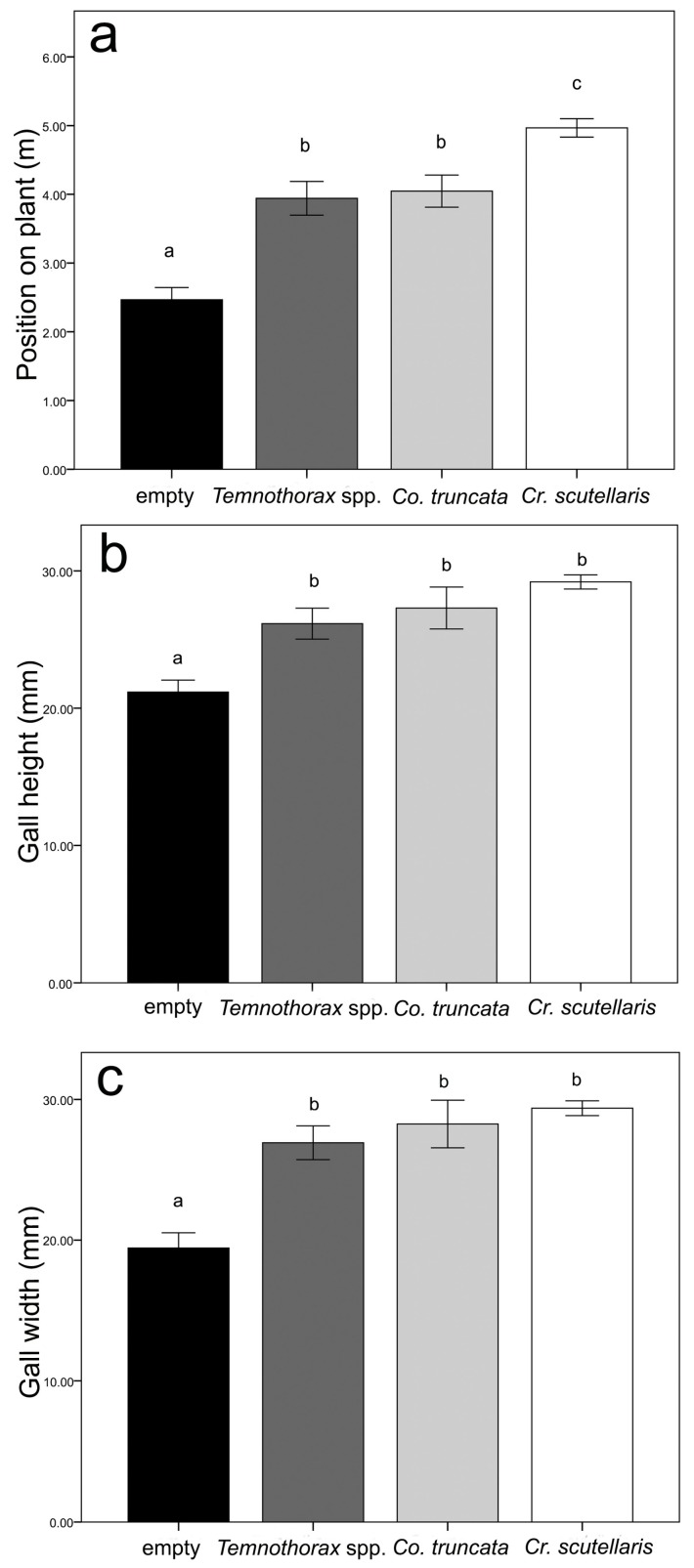
Graphs showing gall preferences of the different ant species according to the data collected in Experiment 1. The gall preferences are measured as gall position on the plant (**a**), gall height (**b**), and gall width (**c**). The SE interval is shown for each bar. The bars with the same letter are not statistically different (One-way ANOVA, see text for further details).

In order to investigate differences between ant species on gall position on the plant and gall size, we excluded the gall categories with less than five samples from the analysis (mouldy galls and galls with *D. quadripunctatus*). In addition, we merged data from the species of the genus *Temnothorax* into the same group (*Temnothorax* spp.). The statistical analyses showed similar trends as the field survey: significant differences in gall size and position on the plant according to ant species were detected (n = 107; Position: F_3,103_ = 28.22 *P* < 0.001; Height: F_3,103_ = 15.90 *P* < 0.001; Width: F_3,103_ = 21.36 *P* < 0.001). As regards the position on the plant, the post-hoc comparisons (Table S2) showed that there were three different groups with the highest values for *Cr. scutellaris*, the lowest values for empty galls, and values for *Temnothorax* spp. and *C. truncata* in between (Figure 3a). With regard to gall height and width, the post-hoc analysis (Table S2) showed that there were two groups: the biggest galls were colonized by *Cr. scutellaris*, *Co. truncata* and *Temnothorax* spp., whereas the smallest ones were empty (Figure 3b,c).

The analysis of colony composition in the galls colonized by *Cr. scutellaris* (n = 69) showed that 23 galls hosted queens only, nine galls a queen, workers and brood (No. of workers: Mean ± SE 65.7 ± 9.7), 28 galls workers and brood (No. of workers: 189.5 ± 25.1), and nine galls workers only (No. of workers: 268.6 ± 27.3) (see Table 1). The galls were divided into two groups for a statistical analysis too: galls with the queen and galls without the queen. The One-way ANOVA tests showed significant differences in gall size according to the presence of the queen (Height: F_1,67_ = 15.71, P < 0.001; Width: F_1,67_ = 9.40, P = 0.003). In particular, the galls with the queen were smaller than the ones without (Mean ± SE - Height: with queen 27.0 ± 0.8 mm, without queen 30.7 ± 0.6 mm; Width: with queen 27.5 ± 0.8 mm, without queen 30.6 ± 0.6 mm). No differences were detected for gall position on the plant (F_1,67_ = 0.90, P = 0.347; Mean ± SE: with queen 4.8 ± 0.2 m, without queen 5.1 ± 0.2 m).

### 3.2. Nest Architecture

**Figure 4:**
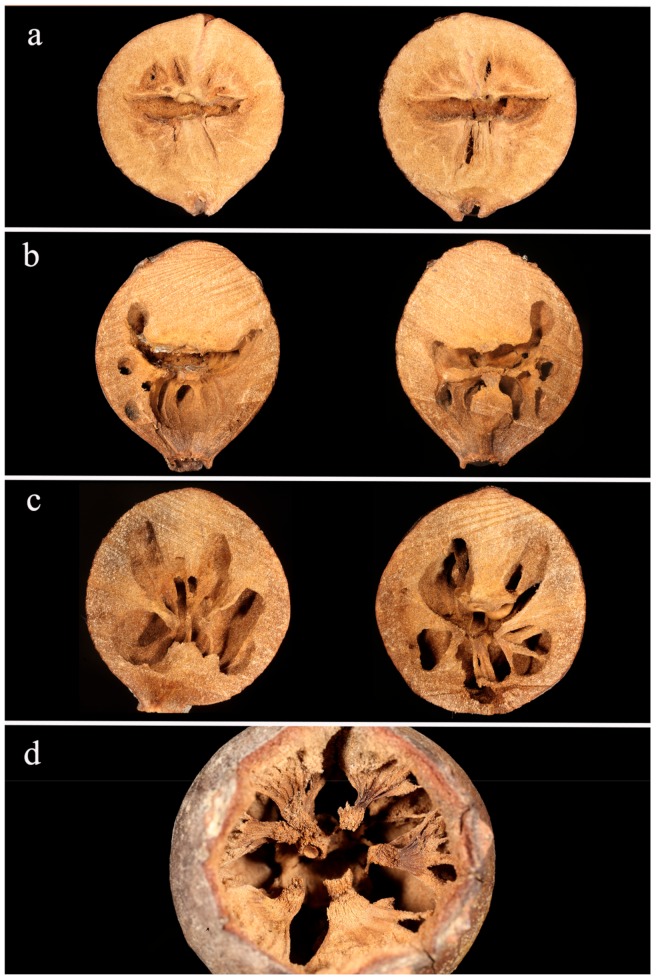
Inner architecture of a gall, when the gall is empty (**a**), colonized by *Co. truncata* (**b**) or by *Temnothorax* spp. (**c**,**d**). Images a, b, c show the two halves of the gall (see text for details). In image d, the gall is seen from above and the upper part has been removed.

**Figure 5:**
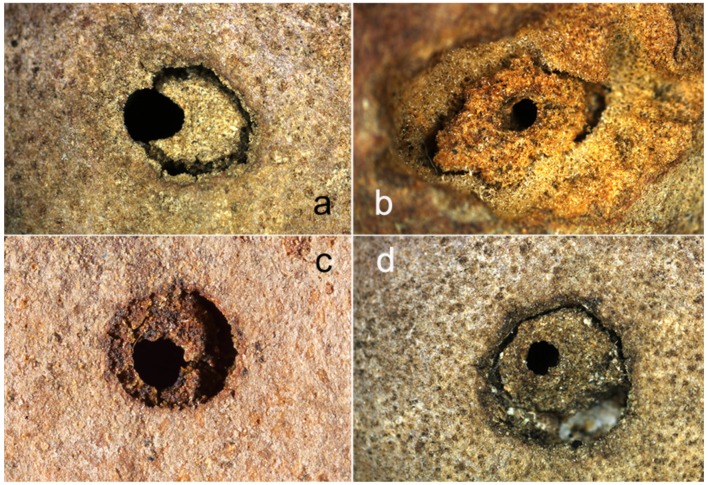
Gall entrance hole modified by *Temnothorax* spp. Pictures (**a**–**d**) show different examples of reduction of entry hole.

**Figure 6:**
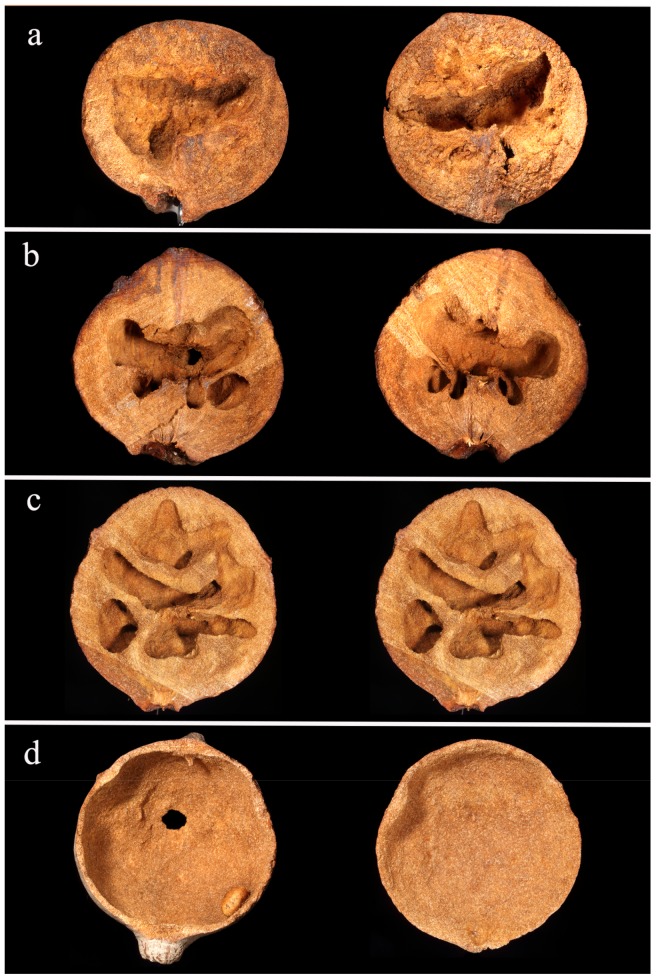
Inner architecture of a gall colonized by *Cr. scutellaris*. The effect of different colony composition is shown: queen only (**a**), queen+workers+brood (**b**), workers+brood (**c**), workers only (**d**). In (**d**), the puparium of the cynipid wasp is still present.

As for the qualitative description of the inner architecture of the galls, the empty galls presented a characteristic elongated air space where the cynipid puparium was located in the middle (Figure 4a). The air space was still visible in the galls occupied by *Co. truncata*, but new excavated areas were in the lower part only. This additional air space was organized into few small radially distributed chambers/galleries. Unlike the other species, the upper half of the gall was not modified. (Figure 4b). In the galls occupied by *Temnothorax* spp., the air space originated by the cynipid was no longer visible because several very evident tunnels crossed all parts of gall (Figure 4c). A radial architecture was found by sectioning the gall according to a perpendicular plane in relation to our cutting procedure with many excavated portions diverging radially from the centre. Visible septa were characterized by different thicknesses and sizes, probably influenced by the compactness of the material composing the gall (Figure 4d). About 80% of the colonized galls (n = 10) presented an obvious reduction of the entrance hole obtained by the ants by adding debris (Figure 5). The galls inhabited by *Cr. scutellaris* presented different internal architecture depending on the ant colony composition. When a founding queen only inhabited the gall, the air space originated by the cynipid was still visible but extended (Figure 6a). When workers and brood were also found alongside a queen, the inner part of the gall presented a central chamber and a number of small sub-chambers. In these cases, the ants excavated the gall in all directions and the original shape of the inner area was no longer visible (Figure 6b). Moreover, when the gall was occupied by workers and brood, the amount of excavated material was even more significant, larger rooms were found and the internal architecture was more complex (Figure 6c). The degree of excavation of the gall reached its maximum in galls where only workers were found. The gall appeared almost completely excavated, with no visible chambers, and the external wall of the gall was very thin (Figure 6d).

**Figure 7:**
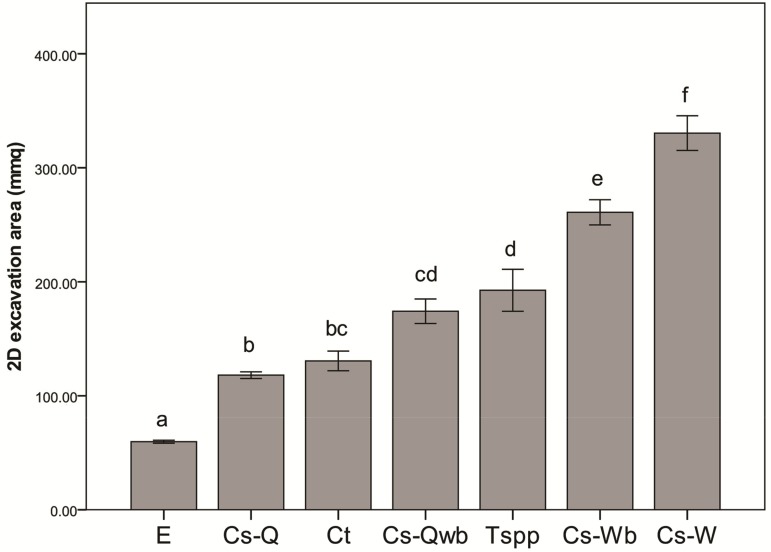
2D excavation area according to ant species and colony composition. E = empty, Cs-Q = *Cr. scutellaris* queen only, Ct = *Co. truncata*, Cs-Qwb = *Cr. scutellaris* queen+workers+brood, Tspp = *Temnothorax* spp., Cs-Wb = *Cr. scutellaris* workers+brood, Cs-W = *Cr. scutellaris* workers only. The SE interval is shown for each bar. The bars with the same letter are not statistically different (One-way ANOVA, see text for further details).

With regard to the analysis of 2D excavation area, the One-way ANOVA test found significant differences between the seven categories (F_6,49_ = 65.53 *P* < 0.001). The post-hoc tests found four different groups (Table S3). The biggest area was related to *Cr. scutellaris*/workers and was followed by *Cr. scutellaris*/workers+brood. Then, there was a complex of *Temnothorax* spp., *Cr. scutellaris/* queen+workers+brood, *Cr. truncata* and *Cr. scutellaris*/queen. The smallest area was recorded for empty galls (Figure 7).

**Figure 8:**
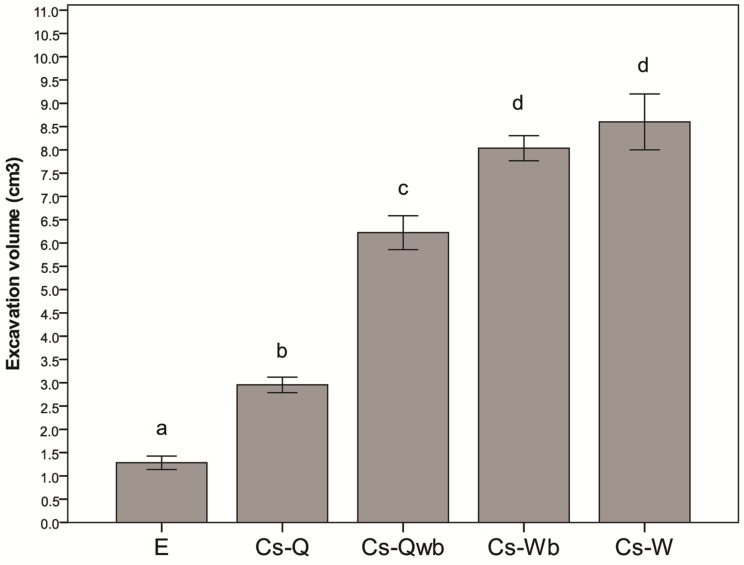
Excavation volume in galls colonized by *Cr. scutellaris* according to colony composition: E = empty, Cs-Q = *Cr. scutellaris* queen only, Cs-Qwb = *Cr. scutellaris* queen+workers+brood, Cs-Wb = *Cr. scutellaris* workers+brood, Cs-W = *Cr. scutellaris* workers only. The SE interval is shown for each bar. The bars with the same letter are not statistically different (One-way ANOVA, see text for further details).

The analysis on excavation volume on galls colonized by *Cr. scutellaris* (n = 85) showed significant differences between the different categories of colony compositions (One-way ANOVA: F_4,80_ = 122.85 *P* < 0.001). Tukey tests showed four different groups with the biggest volumes being associated with galls with workers only and workers+brood (Table S4). The other categories were listed according to the following decreasing gradient: queen+workers+brood, queen only, and empty galls (Figure 8).

### 3.3. Defensive Effect of Ants on Plants: Experiment 2

**Figure 9:**
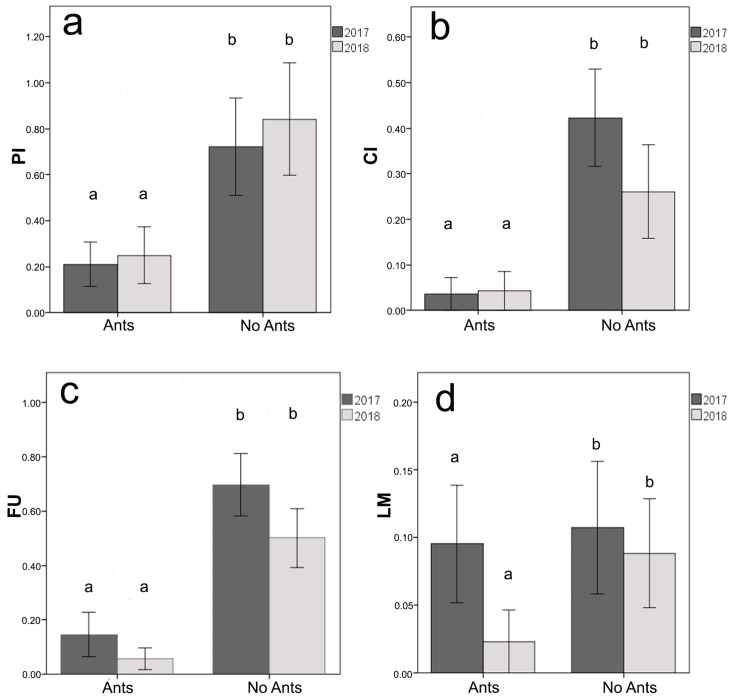
Effects of the ant presence on plant health. The effects are shown according to the two years of sampling (2017–2018). (**a**) PI: mean number of Phytophagous Insects, (**b**) CI: mean proportion of leaves attacked by chewing insects on the total leaves, (**c**) FU: mean proportion of leaves attacked by Fungi on the total leaves, (**d**) LM: mean proportion of leaves attacked by leaf miners on the total leaves. The SE interval is shown for each bar. The bars with the same letter are not statistically different (Two-way ANOVA, see text for further details).

The analysis of the colony composition of the 76 experimental galls showed that the galls initially classified as empty (N = 37) did not host ants, whereas those classified as “with ants” hosted workers only (No. of galls = 21; No. of workers: 207.0 ± 13.6), or workers and brood (No. of galls = 18; No. of workers: 155.1 ± 9.5). The analysis on the presence of phytophagous insects showed that the year had no effect (F_1,72_ = 0.20, *P* = 0.655), whereas the presence of ants did (F_1,72_ = 9.67, *P* = 0.003). The number of phytophagous insects decreased when the ants were present. The interaction between the two factors (year*ants) was not significant (F_1,72_ = 0.05, *P* = 0.821) (Figure 9a). Even the analysis on leaves attacked by chewing insects showed that the year had no effect (F_1,72_ = 1.00, *P* = 0.322), whereas the presence of ants did (F_1,72_ = 15.10, *P* < 0.001). The number of leaves attacked by chewing insects decreased when the ants were present. The interaction between the two factors (year*ants) was not significant (F_1,72_ = 1.18, *P* = 0.281) (Figure 9b). As for leaves attacked by fungi, the results show that the year had no effect (F_1,72_ = 2.54, *P* = 0.115), whereas the presence of ants did (F_1,72_ = 31.04, *P* < 0.001). The number of leaves attacked by fungi decreased when the ants were present. The interaction between the two factors (year*ants) was not significant (F_1,72_ = 0.36, *P* = 0.551) (Figure 9c). As regards the analysis on leaves attacked by leaf-miners, neither the year (F_1,72_ = 1.32, *P* = 0.255) nor the presence of ants (F_1,72_ = 0.96, *P* = 0.333) had any effect. The interaction between the two factors (year*ants) was not significant (F_1,72_ = 0.45, *P* = 0.503) (Figure 9d).

## 4. Discussion

Ant–plant interactions represent a textbook example in insect-plant biology for their variety, complexity and spread [27,28,46]. The present study is a first survey and analysis of the ant colonization of galls induced by oak gall wasp *Andricus quercustozae* in Italy showing the importance of ants as plant defenders mediated by these structures. In this context, gall-inducing wasps act as “ecosystem engineers” providing suitable nest sites allowing an abundant and stable presence of ants on the host plant and consequently addressing a cascade of multitrophic interactions involving the ants, the plant and other insects [12,15] These structures represent suitable nest sites allowing an abundant and stable presence of ants on the host plant. We recorded the presence of 12 ant species belonging to five different genera. The most frequent and abundant species appeared to be *Cr. scutellaris*, followed by *Co. truncata* and several species belonging to the genus *Temnothorax*. Species such as *T. albipennis*, *T. nylanderi*, *T. parvulus* and *T. unifasciatus* were found several times nesting arboreally by exploiting the galls microhabitats directly on the plant. This evidence appears remarkable, as those species were almost exclusively known to nest near the soil surface (including on fallen galls) [47]. On the other hand, *T. italicus*, a very little-studied Italian endemic species, has always been considered closely related to other arboreal-nesting *Temnothorax* [48,49]. In one single sample, we also found specimens of *Cr. scutellaris nigra*. Although its taxonomical status as a subspecies was not further investigated after a first description, our data represent the first Italian record outside Sardinia and Sicily [50,51].

European studies on gall colonization by ants with a specific focus on *Quercus* galls date back to the years 1970–1980. In particular, Tòrossian [20] conducted a study in the woods near Toulouse (France) on galls induced by several cynipids, including *A. quercustozae*, proving *Temnothorax* spp., *D. quadripunctatus* and *Co. truncata* to be prevalent species, followed by a second group composed by *Cr. scutellaris* along with *C. fallax*. In the Iberian Peninsula, Espadaler and Nieves [22] analysed a group of galls which had been gathered from 24 sites and were shown to be induced by three different species of cynipids, including *A. quercustozae*. The authors described a group of widespread colonizing species made up of *Cr. scutellaris, Co. truncata, C. fallax* and *C. lateralis,* followed by *D. quadripunctatus*. They also recorded the presence of a less abundant group consisting of various species of the genera *Temnothorax*. Concerning the present study, although the composition of ant species is similar to the one described in both the European studies, the frequency of colonization is more comparable to the one found in the Iberian Peninsula. This result could be connected to similar biogeographic and climatic conditions influencing the initial composition of the colonizing species [22].

The results of gall colonization by ants provide some indications on this peculiar ant–plant system mediated by these structures. We showed clear gall preferences by the different ant species. All the colonizing species excluded the smallest galls and galls located on the lower part of the plant (1–2 m). This may suggest two interpretations. One is that this may have a significant defensive role for incipient colonies since the galls located further up the tree could suffer fewer attacks or accidental damages, but further studies are needed to test this hypothesis. Another is that larger galls could prove useful in a subsequent “ergonomic phase” of colony growth when the housed population presumably is subject to a large increase [46]. Although referring to a tropical plant, studies on candeia trees (*Eremanthus erythropappus*) showed similar patterns in gall size preferences: larger galls have higher rates of occupation by ants [23,52].

Among colonized galls, we recorded a more evident tendency of *Cr. scutellaris* to occupy larger galls that are located at higher positions on trees (4–5 m from the ground). The latter preference is in accordance with the data on *Cr. scutellaris* collected in France [15]. As far as gall size is concerned, our results could be related to the mean body size of *Cr. scutellaris* workers, which is greater than the other species. In fact, *Cr. scutellaris* workers have a mean length of about 8.8 mm, whereas *Cr. truncata* major workers vary between 5.2 and 6 mm, *Co. truncata* minor workers are usually less than 3.5 mm, and *Temnothorax* spp. workers range between 2 and 3 mm [53,54]. In addition, gall-size preferences could be related to colony size: *Cr. scutellaris* colonies can host up to 5000 individuals, whereas *Co. truncata* and *Temnothorax* spp. colonies reach a maximum of few hundred individuals [54,55]. This pattern of gall occupancy and distribution may result, on the one hand, from an initial species-specific preference for gall features, and on the other, from an exclusion of the other species operated by *Cr. scutellaris.* This species is considered one of the most highly ranked competitors in Mediterranean ant communities and its presence may affect the nesting and performance of other ants [56,57,58,59,60].

The analysis of the colony composition indicated differences in gall size according to the presence of the queen only for *Cr. scutellaris*. Galls with a queen are usually smaller than galls without. Further analyses are needed to investigate possible cues and mechanisms used by founding queens in their choice. We may suppose that queens prefer smaller galls in the early stage of foundation for a matter of greater protection: smaller surfaces are indeed less exposed to mechanical damages or to the action of atmospheric agents; this observation may be further guaranteed by the inner architecture with small rooms and thick walls (see below). On the contrary, galls that hosted brood only and/or workers were larger. This condition could be related to the colony-growing phase when larger spaces are needed and part of the colony transfers to surrounding galls. This is consistent with polydomic habits common in ants and reported for *Cr. scutellaris* too, implying a division of a colony’s section into several nesting sites [59,61,62,63]. An alternative use of galls by ants is that of “outstations” as reported on candeia trees [23]. An outstation is a pre-existing structure in the environment used as a rest area or a shelter during territory patrolling, eventually enabling the ants to respond to nest invasions/disturbances quickly [64,65].

The polydomic habits during colony growth in *Cr. scutellaris* can also explain differences in gall inner architecture in connection with the presence of the queen. In fact, galls hosting a queen revealed smaller volumes of removed material compared to the queen-free galls. This probably depends on an optimization of resources operated by the queen, which requires less space, while workers carry out more intense excavation to accommodate a growing number of individuals and resources. Moreover, the complexity of the inner architecture (presence of rooms and corridors) was greater in the galls that hosted different categories of individuals (queen + workers + brood, or workers + brood), whereas the architecture in galls with the queen only (poorly excavated) or workers only (fully excavated) was simpler. It is worth pointing out that we never found queens in galls with a large number of workers and brood. Based on data, we can speculate that there are typical “queen galls” that are occupied by the queen and her incipient colony from the beginning, then by brood and young workers as they are produced. Later, most of the worker population migrates to other galls, thereby deeply modifying their architecture as they grow in age and number, as happens in some ground-dwelling ants [66,67].

The comparison of the inner architecture between species showed that *Cr. scutellaris* was the only one in which the internal nest features were related to colony composition, while the other species presented only one characteristic structure. *Co. truncata* tended to excavate only half of the gall, whereas *Temnothorax* spp. extended the excavation to the entire gall. However, in both cases, chambers and tunnels were clearly visible so that the inner architecture showed the highest complexity. Varoudis et al. (2018) [68] found a similar trend in their study on nest structures of acorn-dwelling ants using X-ray microtomography. In this study, they highlighted that nests of *Temnothorax* spp. showed highly compartmentalized architectural elements and discrete zones of connectivity. As for other adjustments in gall structure, our study showed that only *Temnothorax* spp. modified the nest entrance, reducing the dimension of the opening hole, probably to limit intrusions and guarantee a selective entrance into the colony. The role of different types of inner architecture in gall-nesting ants could be a key factor in ant migration and relocation in response to colony growth or environmental variables (such as temperature or pathogen exposure), as noted for the acorn ant *Temnothorax curvispinosus* [69].

Our analysis on the effects of the ants on plant health proves a positive effect on leaf damages and a decrease in phytophagous insects due to the ant presence. This result is consistent with the positive impact of ants on plant predators reported in many field and laboratory studies [24,25,70,71,72,73,74]. The defensive outcome may follow from the deterrent effect of ant presence and patrolling on other insects, or from direct attacks on phytophagous arthropods or their eggs [28,75]. Interestingly, the positive effect of ants in reducing the number of leaves attacked by fungi could be linked to both a mechanical removal or the powerful antifungal chemicals used by ants and dispersed during the patrolling activity [27,46]. The defence against pathogens could also be achieved through the presence of specific bacterial communities on the ant legs [76]. In general, the ability of the ants to modify their micro-habitats and limit the pathogens in their nest or feeding area is a key to their ecological success, and this could be indirectly beneficial for the plants that are able to host these insects [28,77,78].

As for the impact of galls on plants, several studies have demonstrated that gall-inducing insects are considered parasites which produce deleterious effects on the growth and fitness (i.e., a reduction in flowers, fruits, seeds, leaves, shoots, and biomass production) of their host plant ([19] and references within). Besides, little research has highlighted the mutualistic relationship between gallers and their host plants, such as, for example, studies on agaonid fig wasps and prodoxine yucca moths. In these two cases, gallers also represent highly specific pollen vectors, showing behavioural and/or morphological adaptations to enhance pollination effectiveness [79]. Our research underlines a special feature of this peculiar ant–plant relationship: being permanently housed in the galls, ants have a beneficial effect on the plant. From this point of view, galls can be considered as having the same function as “domatia” produced by myrmecophyte plants as special structures allowing the settlement of ant colonies on the plant and the subsequent benefits [24,27,72,80]. Hence, galls can be seen as the result of a plant parasite action that ultimately produces a potentially beneficial “side” effect for the host plant.

## 5. Conclusions

Our study suggests that ants hosted by galls efficiently protect their hosting plants against enemies (predators and pathogens), even though galls are initially a potential cost for the plant. This represents a new and more complex multipartite system involving insects and plants with beneficial effects on the plants as by-product of a costly situation. To our knowledge, no increased fitness in galled oak plants due to secondary colonization has been demonstrated to date [81]. Galls are a problem for the plant but following ant colonization, they could become a resource providing also benefits. Therefore, we can speculate that gall-dwelling ants may affect (or have affected) the (co)evolutionary dynamics involving plants and their parasites. This paradigm needs further investigation and offers new perspectives in the reconstruction of the evolutionary pathways and selective pressures that shaped the complex network involving gall-inducers, plants and ants.

## Figures and Tables

**Table 1:** Checklist of the ant species collected during the field survey and Experiment 1. The first column (Tot) shows the total number of galls collected for each species. In the Colony Composition section, the number of galls is shown according to the presence of the queen only (Q), queen+workers+brood (QWB), workers+brood (WB), and workers (W) only.

Species	Field Survey					Field Experiment 1				
	Tot	Colony Composition				Tot	Colony Composition			
		Q	QWB	WB	W		Q	QWB	WB	W
*Crematogaster scutellaris* (Olivier, 1792)	39	3	14	19	3	69	23	9	28	9
*Colobopsis truncata* (Spinola, 1808)	9	-	1	-	8	12	3	-	-	9
*Temnothorax albipennis* (Curtis, 1854)	6	-	6	-	-	7	-	7	-	-
*Temnothorax unifasciatus* (Latreille, 1798)	2	-	2	-	-	1	-	1	-	-
*Temnothorax italicus* (Consani,1952)	10	-	10	-	-	2	-	2	-	-
*Dolichoderus quadripunctatus* (Linneus, 1771)	4	-	-	-	4	3	-	-	-	3
*Camponotus fallax* (Nylander, 1856)	1	-	1	-	-	-	-	-	-	-
*Camponotus lateralis* (Olivier, 1792)	1	-	1	-	-	-	-	-	-	-
*Crematogaster scutellaris nigra* (Krausse, 1912)	1	-	-	-	1	-	-	-	-	-
*Temnothorax nylanderi* (Foerster, 1850)	1	-	1	-	-	-	-	-	-	-
Total	74					94				

## Supplementary Materials

The following are available online at https://www.mdpi.com/2075-4450/10/11/392/s1, Table S1: Multiple comparisons (Tukey test) for Field survey in order to investigate differences between ant species in gall colonization, Table S2: Multiple comparisons (Tukey test) for Experiment 1 in order to investigate after 1 year differences between ant species in gall colonization, Table S3: Multiple comparisons (Tukey test) for 2D excavation area (see text for details) according to ant species and colony composition, Table S4: Multiple comparisons (Tukey test) to evaluate differences on excavation volume (see text for details) according to colony composition on galls colonized by Cr. scutellaris.
