# Supplementary material for: Gall-Colonizing Ants and Their Role as Plant Defenders: From ’Bad Job’ to ’Useful Service’
Source: Insects. 2019 Nov 6;10(11):392. doi: 10.3390/insects10110392 (PMC6920797; doi:10.3390/insects10110392)
Supplement: Supplementary file 1 [file insects-10-00392-s001.pdf]

Table S1. Multiple comparisons (Tukey test) for Field survey in order to investigate differences between ant species in gall colonization. One-Way ANOVA tests were conducted for the three variables measured: 1. position on the plant; 2. gall height; 3. gall width.

		(I) species	(J) species	Difference between mean value (J-I)	Std. Er	Sig
**Position on plant**	Tukey test	*Cr. scutellaris*	*Temnothorax* spp.	2.02795*	0.26213	< 0.001
			Co. truncata	0.20017	0.34648	0.938
			empty	2.88604*	0.25360	< 0.001
		Temnothorax spp.	*Cr. scutellaris*	−2.02795*	0.26213	< 0.001
			Co. truncata	−1.82778*	0.37913	< 0.001
			empty	0.85810*	0.29666	0.025
		Co. truncata	*Cr. scutellaris*	−0.20017	0.34648	0.938
			Temnothorax spp.	1.82778*	0.37913	< 0.001
			empty	2.68587*	0.37328	< 0.001
		Empty	*Cr. scutellaris*	−2.88604*	0.25360	< 0.001
			Temnothorax spp.	−0.85810*	0.29666	0.025
			Co. truncata	−2.68587*	0.37328	< 0.001
**Gall height**	Tukey test	*Cr. scutellaris*	*Temnothorax* spp.	3.72387*	1.07743	0.005
			Co. truncata	−1.05718	1.42414	0.880
			empty	5.44377*	1.04236	< 0.001
		Temnothorax spp.	*Cr. scutellaris*	−3.72387*	1.07743	0.005
			Co. truncata	−4.78105*	1.55835	00.015
			empty	1.71990	1.21935	0.496
		Co. truncata	*Cr. scutellaris*	1.05718	1.42414	0.880
			Temnothorax spp.	4.78105*	1.55835	0.015
			empty	6.50095*	1.53431	< 0.001
		Empty	*Cr. scutellaris*	−5.44377*	1.04236	< 0.001
			Temnothorax spp.	−1.71990	1.21935	0.496
			Co. truncata	−6.50095*	1.53431	< 0.001
Gall width	Tukey test	*Cr. scutellaris*	*Temnothorax* spp.	3.54618*	1.00913	0.004
			Co. truncata	−1.50709	1.33386	0.672
			empty	7.47179*	.97628	< 0.001
		Temnothorax spp.	*Cr. scutellaris*	−3.54618*	1.00913	.004
			Co. truncata	−5.05327*	1.45957	00.005
			empty	3.92561*	1.14205	0.005
		Co. truncata	*Cr. scutellaris*	1.50709	1.33386	0.672
			Temnothorax spp.	5.05327*	1.45957	0.005
			empty	8.97889*	1.43705	< 0.001
		Empty	*Cr. scutellaris*	−7.47179*	0.97628	< 0.001
			Temnothorax spp.	−3.92561*	1.14205	0.005
			Co. truncata	−8.97889*	1.43705	< 0.001

Table 2. Comparisons (Tukey test) for Experiment 1 in order to investigate after 1 year differences between ant species in gall colonization. One-Way ANOVA tests were conducted for the three variables measured: 1. position on the plant; 2. gall height; 3. gall width.

	Tukey test	(I) species	(J)species	Difference between mean value(J-I)	Std. Er	Sig
Position on plant		*Cr. scutellaris*	*Temnothorax* spp.	1.02452*	0.34018	0.017
			Co. truncata	0.91986*	0.31445	0.022
			empty	2.50027*	0.27896	< 0.001
		Temnothorax spp.	*Cr. scutellaris*	−1.02452*	0.34018	0.017
			Co. truncata	−0.10467	0.43047	0.995
			Empty	1.47575*	0.40527	0.002
		Co. truncata	*Cr. scutellaris*	−0.91986*	0.31445	0.022
			Temnothorax spp.	0.10467	0.43047	0.995
			empty	1.58042*	0.38393	< 0.001
		Empty	*Cr. scutellaris*	−2.50027*	0.27896	< 0.001
			Temnothorax spp.	−1.47575*	0.40527	0.002

			Co. truncata	−1.58042*	0.38393	< 0.001
Gall height	Tukey test	*Cr. scutellaris*	*Temnothorax* spp.	3.03822	1.43435	0.154
			Co. truncata	1.90188	1.32585	0.481
			empty	8.02084*	1.17623	< 0.001
		Temnothorax spp.	*Cr. scutellaris*	−3.03822	1.43435	0.154
			Co. truncata	−1.13633	1.81504	0.923
			empty	4.98262*	1.70881	0.022
		Co. truncata	*Cr. scutellaris*	−1.90188	1.32585	0.481
			Temnothorax spp.	1.13633	1.81504	0.923
			empty	6.11896*	1.61881	0.001
		Empty	*Cr. scutellaris*	−8.02084*	1.17623	< 0.001
			Temnothorax spp.	−4.98262*	1.70881	0.022
			Co. truncata	−6.11896*	1.61881	0.001
Gall width	Tukey test	*Cr. scutellaris*	*Temnothorax* spp.	2.45606	1.51872	0.374
			Co. truncata	1.11822	1.40384	0.856
			empty	9.93093*	1.24542	< 0.001
		Temnothorax spp.	*Cr. scutellaris*	−2.45606	1.51872	0.374
			Co. truncata	−1.33783	1.92181	0.898
			empty	7.47487*	1.80933	< 0.001
		Co. truncata	*Cr. scutellaris*	−1.11822	1.40384	0.856
			Temnothorax spp.	1.33783	1.92181	0.898
			empty	8.81271*	1.71403	< 0.001
		Empty	*Cr. scutellaris*	−9.93093*	1.24542	< 0.001
			Temnothorax spp.	−7.47487*	1.80933	< 0.001
			Co. truncata	−8.81271*	1.71403	< 0.001

Table S3. Multiple comparisons (Tukey test) for 2D excavation area (see text for details) according to ant species and colony composition. One-Way ANOVA tests were run to compare excavation area in relation to species colonization and colony composition.

			ant species and colony composition	Difference between mean value(J-I)	Std. Er	Sig
**Tukey test**	Empty		*Cr. scutellaris*-queen only	−58.39338*	15.96279	0.010
			Cr. scutellaris-queen+workers+brood	−114.45363*	15.96279	< 0.001
			Cr. scutellaris-workers+brood	−201.19663*	15.96279	< 0.001
			Cr. scutellaris-workers only	−270.68425*	15.96279	< 0.001
			Co. truncata	−70.84713*	15.96279	0.001
			Temnothorax spp.	−132.83038*	15.96279	< 0.001
**Tukey test**	*Cr. scutellaris*	queen only	empty	58.39338*	15.96279	0.010
			Cr. scutellaris-queen+workers+brood	−56.06025*	15.96279	0.016
			Cr. scutellaris-workers+brood	−142.80325*	15.96279	< 0.001
			Cr. scutellaris-workers only	−212.29087*	15.96279	< 0.001
			Co. truncata	−12.45375	15.96279	0.986
			Temnothorax spp.	−74.43700*	15.96279	< 0.001
Tukey test	*Cr. scutellaris*	queen+ workers+brood	empty	114.45363*	15.96279	< 0.001
			Cr. scutellaris-queen only	56.06025*	15.96279	0.016
			Cr. scutellaris-workers+brood	−86.74300*	15.96279	< 0.001
			Cr. scutellaris-workers only	−156.23062*	15.96279	< 0.001
			Co. truncata	43.60650	15.96279	0.112
			Temnothorax spp.	−18.37675	15.96279	0.909
Tukey test	*Cr.scutellaris*	workers+brood	empty	201.19663*	15.96279	< 0.001
			Cr. scutellaris-queen only	142.80325*	15.96279	< 0.001
			Cr. scutellaris-queen+workers+brood	86.74300*	15.96279	< 0.001
			Cr. scutellaris-workers only	−69.48762*	15.96279	0.001
			Co. truncata	130.34950*	15.96279	< 0.001
			Temnothorax spp.	68.36625*	15.96279	0.002

Tukey test	*Cr. scutellaris*	workers only	empty	270.68425*	15.96279	< 0.001
			Cr. scutellaris-queen only	212.29087*	15.96279	< 0.001
			Cr. scutellaris-queen+workers+brood	156.23062*	15.96279	< 0.001
			Cr. scutellaris-workers+brood	69.48762*	15.96279	0.001
			Co. truncata	199.83713*	15.96279	< 0.001
			Temnothorax spp.	137.85388*	15.96279	< 0.001
Tukey test	*Co. truncata*		empty	70.84713*	15.96279	0.001
			Cr. scutellaris-queen only	12.45375	15.96279	0.986
			Cr. scutellaris-queen+workers+brood	−43.60650	15.96279	0.112
			Cr. scutellaris-workers+brood	−130.34950*	15.96279	< 0.001
			Cr. scutellaris-workers only	−199.83713*	15.96279	< 0.001
			Temnothorax spp.	−61.98325*	15.96279	0.005
Tukey test	*Temnothorax* spp.		empty	132.83038*	15.96279	< 0.001
			Cr. scutellaris-queen only	74.43700*	15.96279	< 0.001
			Cr. scutellaris-queen+workers+brood	18.37675	15.96279	0.909
			Cr. scutellaris-workers+brood	−68.36625*	15.96279	0.002
			Cr. scutellaris-workers only	−137.85388*	15.96279	< 0.001
			Co. truncata	61.98325*	15.96279	0.005

Table S4. Multiple comparisons (Tukey test) to evaluate differences on excavation volume (see text for details) according to colony composition on galls colonized by *Cr. scutellaris*.

			Cr. Scutellaris colony composition	Difference between mean value(J-I)	Std. Er	Sig
**Tukey test**	*Cr. scutellaris*	queen only	*Cr. scutellaris*-queen+workers+brood	−3.268*	0.474	< 0.001
			Cr. scutellaris-workers+brood	−5.081*	0.341	< 0.001
			Cr. scutellaris-workers only	−5.645*	0.457	< 0.001
			empty	1.673*	0.394	0.001
**Tukey test**	*Cr. scutellaris* workers+brood	queen+	*Cr. scutellaris*-queen only	3.268*	0.474	< 0.001
			Cr. scutellaris-workers+brood	−1.813*	0.459	0.002
			Cr. scutellaris-workers only	−2.378*	0.551	< 0.001
			empty	4.941*	0.499	< 0.001
Tukey test	*Cr. scutellaris*	workers+brood	*Cr. scutellaris*-queen only	5.081*	0.341	< 0.001
			Cr. scutellaris-queen+workers+brood	1.813*	0.459	0.002
			Cr. scutellaris-workers only	−0.564	0.441	0.705
			empty	6.754*	0.376	< 0.001
Tukey test	*Cr. scutellaris*	workers only	*Cr. scutellaris*-queen only	5.645*	0.457	< 0.001
			Cr. scutellaris-queen+workers+brood	2.378*	0.551	< 0.001
			Cr. scutellaris-workers+brood	0.564	0.441	0.705
			empty	7.319*	0.483	< 0.001
Tukey test	empty		*Cr. scutellaris*-queen only	−1.673*	0.394	0.001
			Cr. scutellaris-workers+brood	−4.941*	0.499	< 0.001
			Cr. scutellaris-workers+brood	−6.754*	0.376	< 0.001
			Cr. scutellaris-workers only	−7.319*	0.483	< 0.001
